# Thyroid redox imbalance in adult Wistar rats that were exposed to nicotine during breastfeeding

**DOI:** 10.1038/s41598-020-72725-w

**Published:** 2020-09-24

**Authors:** Rosiane Aparecida Miranda, Egberto Gaspar de Moura, Patrícia Novaes Soares, Thamara Cherem Peixoto, Bruna Pereira Lopes, Cherley Borba Vieira de Andrade, Elaine de Oliveira, Alex C. Manhães, Caroline Coelho de Faria, Rodrigo Soares Fortunato, Patricia Cristina Lisboa

**Affiliations:** 1 Physiological Sciences Department, Laboratory of Endocrine Physiology, Biology Institute, Rio de Janeiro State University, Avenida 28 de Setembro, 87, Rio de Janeiro, RJ 20551-031 Brazil; 2 Translational Endocrinology Laboratory, Carlos Chagas Filho Biophysics Institute, Federal University of Rio de Janeiro, Rio de Janeiro, Brazil; 3 Laboratory of Neurophysiology, Biology Institute, Rio de Janeiro State University, Rio de Janeiro, RJ Brazil; 4 Laboratory of Molecular Radiobiology, Carlos Chagas Filho Biophysics Institute, Federal University of Rio de Janeiro, Rio de Janeiro, Brazil

**Keywords:** Physiology, Metabolism, Hormones

## Abstract

Maternal nicotine exposure causes several consequences in offspring phenotype, such as obesity and thyroid dysfunctions. Nicotine exposure can increase oxidative stress levels, which could lead to thyroid dysfunction. However, the mechanism by which nicotine exposure during breastfeeding leads to thyroid gland dysfunction remains elusive. We aimed to investigate the long-term effects of maternal nicotine exposure on redox homeostasis in thyroid gland, besides other essential steps for thyroid hormone synthesis in rats from both sexes. Lactating Wistar rats were implanted with osmotic minipumps releasing nicotine (NIC, 6 mg/kg/day) or saline (control) from postnatal day 2 to 16. Offspring were analyzed at 180-day-old. NIC males showed lower plasma TSH, T_3_ and T_4_ while NIC females had higher T_3_ and T_4_. In thyroid, NIC males had higher sodium-iodide symporter protein expression, whereas NIC females had higher thyroid-stimulating hormone receptor (TSHr) and thyroperoxidase (TPO) protein expression. TPO activity was lower in NIC males. Hydrogen peroxide generation was decreased in NIC males. Activities of superoxide dismutase, catalase and glutathione peroxidase were compromised in NIC animals from both sexes. 4-Hydroxynonenal was higher only in NIC females, while thiol was not affected in NIC animals from both sexes. NIC offspring also had altered expression of sex steroid receptors in thyroid gland. Both sexes showed similar thyroid morphology, with lower follicle and colloid size. Thyroid from female offspring exposed to nicotine during breastfeeding developed oxidative stress, while the male gland seemed to be protected from redox damage. Thyroid dysfunctions seem to be associated with redox imbalance in a sex-dependent manner.

## Introduction

Early life insults are intimately associated with endocrine and metabolic disorders, such as hypo- and/or hyperthyroidism, diabetes mellitus, cardiovascular diseases, obesity, among others. The hypothesis that relates the perinatal origins with adult diseases is named “Developmental origins of health and diseases” (DOHaD)^1^. This concept includes any factor that could impact the development during phases of great vulnerability, like gestation and lactation. Environmental changes, such as diet, stress, chemical exposures and drugs were shown to program the progeny to serious disturbances at adulthood^2,3^. For instance, children from parents who smoke are more susceptible to non-communicable diseases later in life^4^.

Nicotine is one of the thousands of components of cigarette that potentially cause long-term adverse effects to infants via breast milk^5^. Among these disturbances, we will highlight here those associated with the development of thyroid diseases^6–8^. As already demonstrated by our group, maternal nicotine exposure in rats during breastfeeding induces short- and long-term hypothyroidism associated with thyroid dysfunctions in male offspring^9–11^. The thyroid dysfunction involves several mechanisms including those related to an increased oxidative stress status. However, the impact of nicotine exposure during breastfeeding on oxidative stress remains elusive.

Thyroid hormonogenesis is a complex process that requires multistep reactions that depend on iodide (I^−^) uptake and hydrogen peroxide (H_2_O_2_) production. In the thyrocytes, I^−^ is transported by the sodium-iodide symporter (NIS) located in the basolateral membrane. At the apical membrane, thyroperoxidase (TPO) catalyzes I^−^ oxidation using H_2_O_2_ as cofactor, which is produced by the dual oxidase 2 (DUOX2)^12^. Then, TPO catalyzes the iodination of tyrosine in the thyroglobulin (TG) and then couple iodotyrosyl residues forming the thyroid hormones: l-3,5,3′-triiodothyronine (T_3_) and l-3,5,3′,5′-tetraiodothyronine (T_4_)^13^. T_3_ and T_4_ are then released to circulation through monocarboxylate transporters (MCT). Although H_2_O_2_ generation is crucial to thyroid hormones synthesis, an excess in its production and/or an insufficient protection by intracellular first line antioxidant defenses could compromise the cells integrity leading to thyroid dysfunctions^14^.

Even though studies have demonstrated a relationship between thyroid pathologies and thyrocytes redox imbalance^15–17^, the interconnection between metabolic programming and oxidative stress in the thyroid is mostly unknown. Our hypothesis is that maternal exposure to nicotine during breastfeeding impact directly the redox homeostasis of the thyroid gland, impairing the thyroid hormonogenesis process in the offspring and imprinting thyroid dysfunctions later in life. In addition, we also hypothesized that these dysfunctions would occur in a sex dependent manner.

## Results

### Biometric parameters and hormonal analyses

Nicotine exposure during lactation increased the body mass of male offspring at adulthood; female body mass was not significantly affected. We did not observe changes in absolute or relative thyroid mass in both sexes (Table 1).

**Table 1:** Effect of nicotine exposure during lactation on biometric and hormonal parameters in both male and female rat offspring at 180-day-old.

Parameters	Males		Females	
	Control	Nicotine	Control	Nicotine
Body mass (g)	543.0 ± 7.6	567.8 ± 8.4*	296.1 ± 4.6	292.7 ± 4.4
Absolute thyroid weight (mg)	22.8 ± 1.8	20.4 ± 1.3	20.8 ± 1.5	19.2 ± 1.0
Relative thyroid weight (mg/100 g BM)	4.23 ± 0.36	3.77 ± 0.20	7.08 ± 0.53	6.81 ± 0.41
TSH (ng/ml)	3.68 ± 0.17	3.10 ± 0.05*	3.74 ± 0.16	3.59 ± 0.21
Total T_3_ (ng/dl)	83.6 ± 10.7	48.1 ± 8.6*	84.6 ± 12.2	135.9 ± 8.6*
Free T_4_ (ng/dl)	1.25 ± 0.11	0.93 ± 0.06*	0.78 ± 0.04	1.07 ± 0.11*
Testosterone (ng/ml)	8.67 ± 1.11	5.97 ± 0.42*	0.76 ± 0.12	0.69 ± 0.10
Estradiol (pg/ml)	102.6 ± 7.3	100.7 ± 13.4	149.5 ± 15.3	145.1 ± 13.0

*p < 0.05 vs control group, based on Student’s *t* test. The data represent the mean ± SEM obtained from nine animals from different litters/group (plasma analyses).

*BM* body mass.

NIC males had lower plasma TSH, T_3_ and T_4_ (− 15%, − 42% and − 25%, respectively, *p* < 0.05), while NIC females had higher T_3_ (+ 60%, *p* = 0.004) and T_4_ (+ 37%, *p* = 0.03) (Table 1). We did not observe changes in estradiol levels in both sexes. On the other hand, NIC males displayed lower testosterone levels (− 31%, *p* = 0.04) when compared to control ones, without differences in females (Table 1).

### NIS, TPO, MCT8 and TSHr protein expression

**Figure 1:**
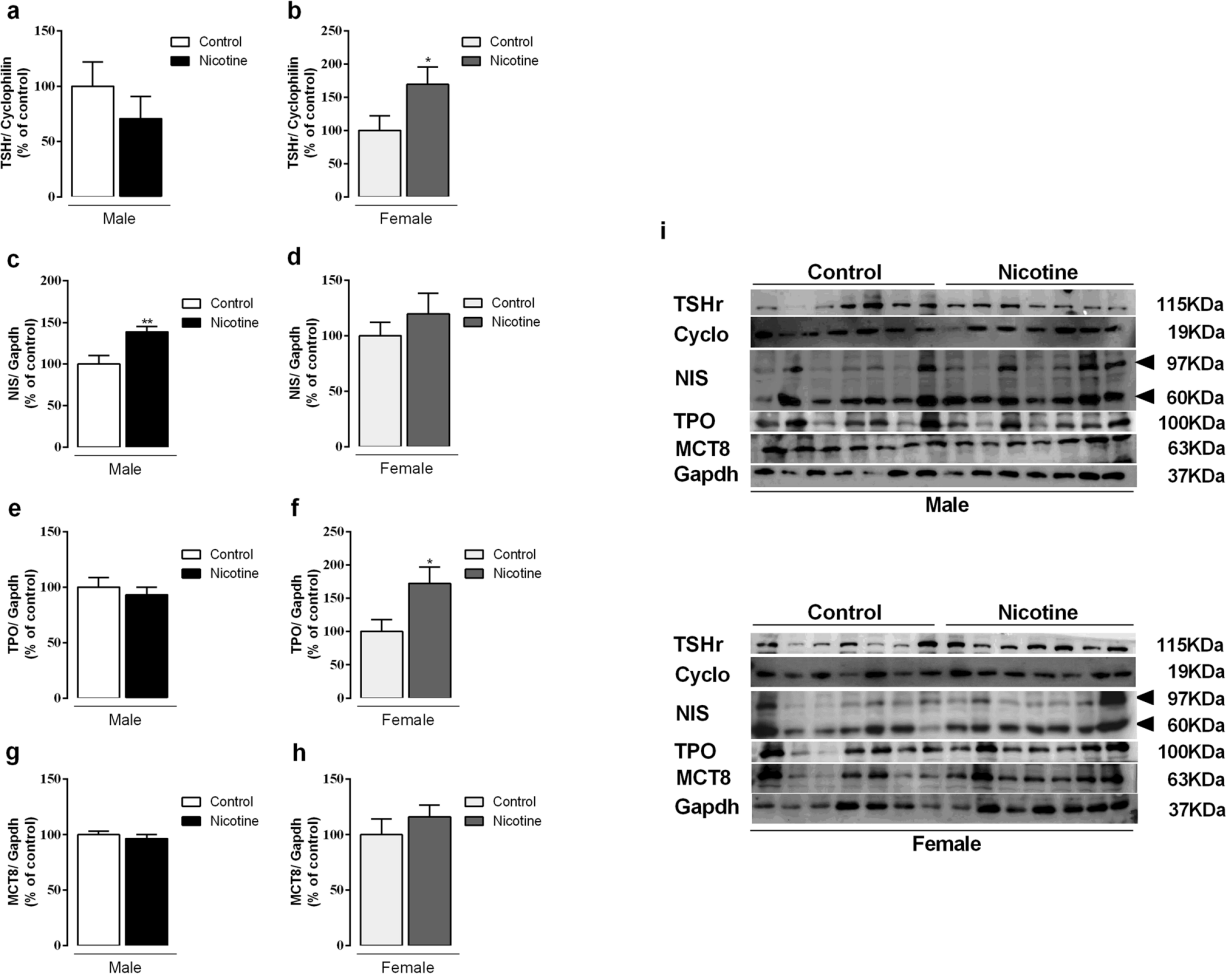
Effects of nicotine exposure during breastfeeding on thyroid-stimulating hormone receptor (TSHr) (**a**, **b**), thyroid sodium-iodide symporter (NIS) (**c**, **d**), thyroperoxidase (TPO) (**e**, **f**), and monocarboxylate transporter 8 (MCT8) (**g**, **h**) protein expression in both male and female offspring at 180-day-old. Representative western blots images show all bands and cropped membrane in specific molecular weight (see Supplementary information) (**i**). Data are expressed as mean ± S.E.M, n = 7 animals from different litters/group; *p < 0.05, **p < 0.001.

In the thyroid, we observed higher NIS (+ 38%, *p* = 0.007) and no changes in TSHr and TPO protein expression in NIC males (Fig. 1a,c,e). In contrast, NIC females had higher TSHr (+ 70%, *p* = 0.03) and TPO protein expression (+ 72%, *p* = 0.03), without differences in NIS (Fig. 1b,d,f). MCT8 protein expression was not affected in both sexes (Fig. 1g,h). Representative western blot images are depicted in Fig. 1i.

### TPO and NOX activity

**Figure 2:**
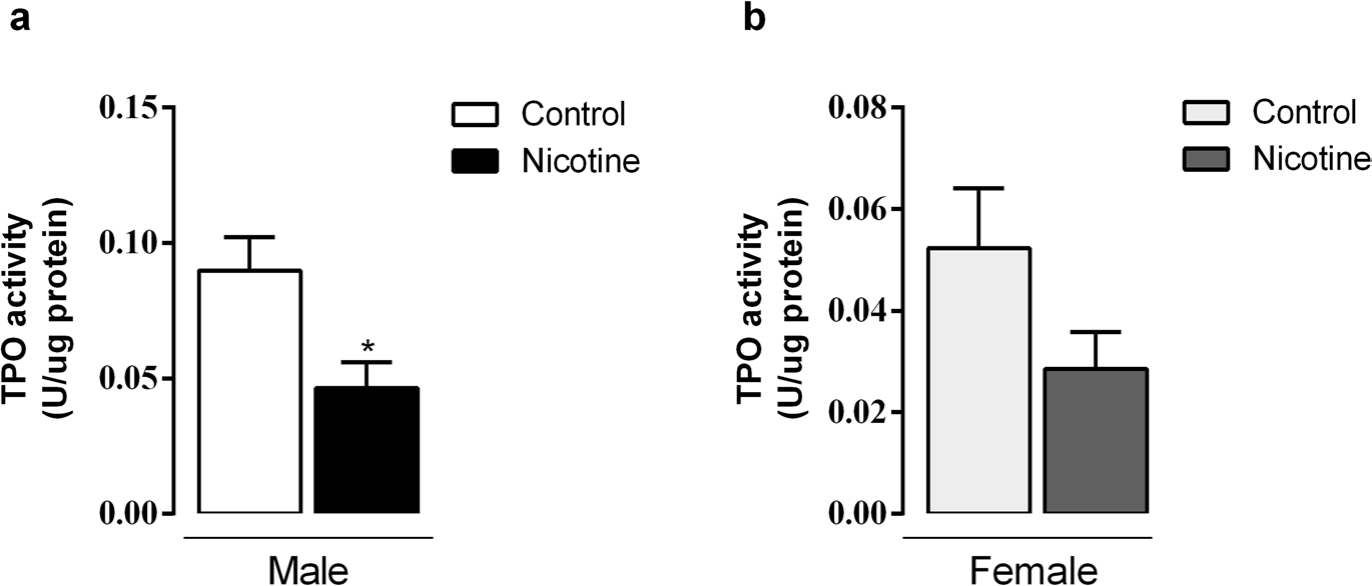
Effects of nicotine exposure during breastfeeding on in vitro thyroid TPO activity of both male (**a**) and female (**b**) offspring at 180-day-old. Data are expressed as mean ± S.E.M, n = 6 animals from different litters/group; *p < 0.05.

**Figure 3:**
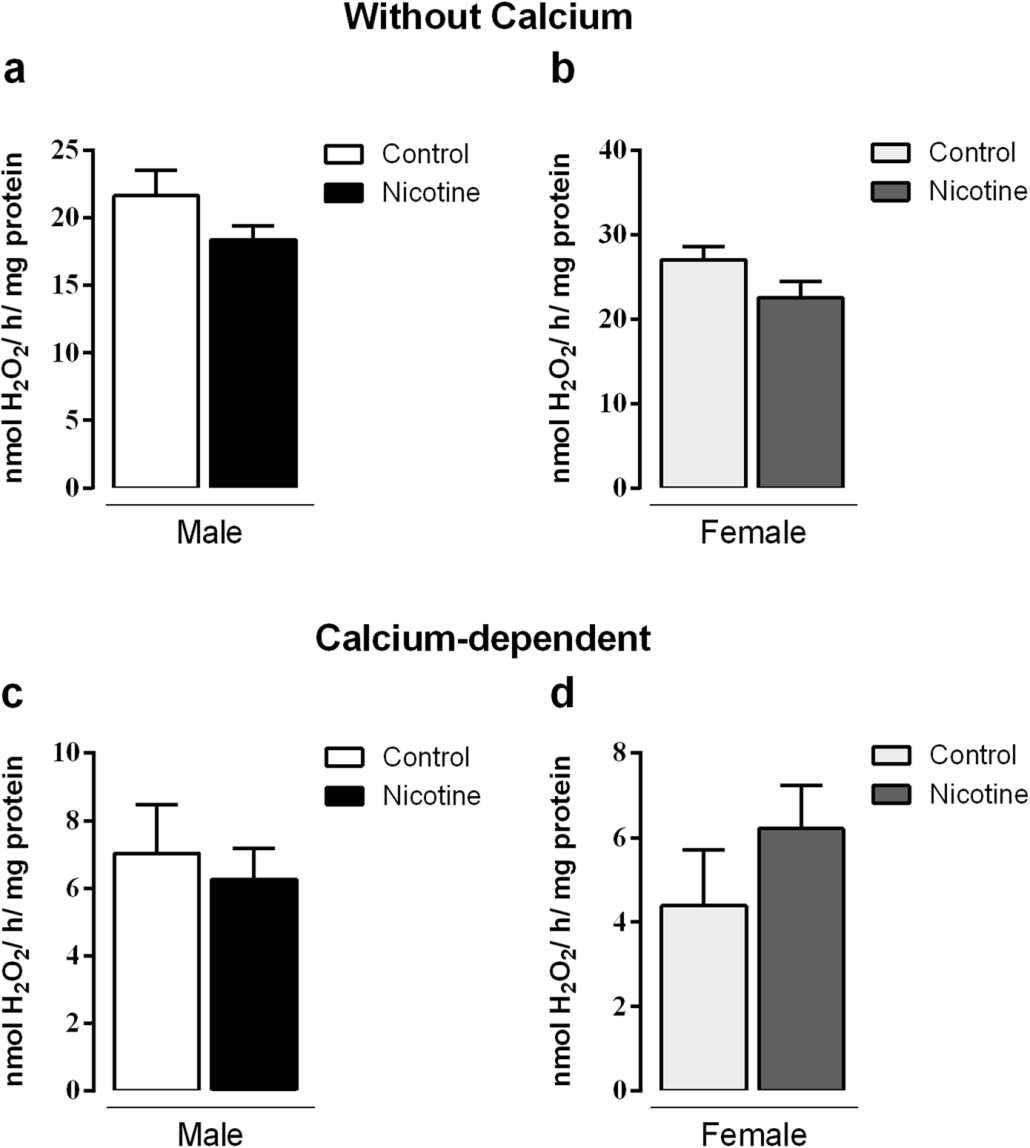
Effects of nicotine exposure during breastfeeding on thyroid microsomal fraction NOX activity without (**a**, **b**) and with calcium (**c**, **d**) in both male and female offspring at 180-day-old. Data are expressed as mean ± S.E.M, n = 6 animals from different litters/group; *p < 0.05.

Thyroid TPO activity in NIC males decreased by -48% (*p* = 0.01, Fig. 2a). NIC females also had decreased TPO activity, despite non-significant statistical difference (Fig. 2b). Thyroid H_2_O_2_ generation in absence of calcium (NOX activity) and calcium-dependent (DUOX activity) did not change in all groups (Fig. 3a–d).

### Antioxidant enzymes

**Figure 4:**
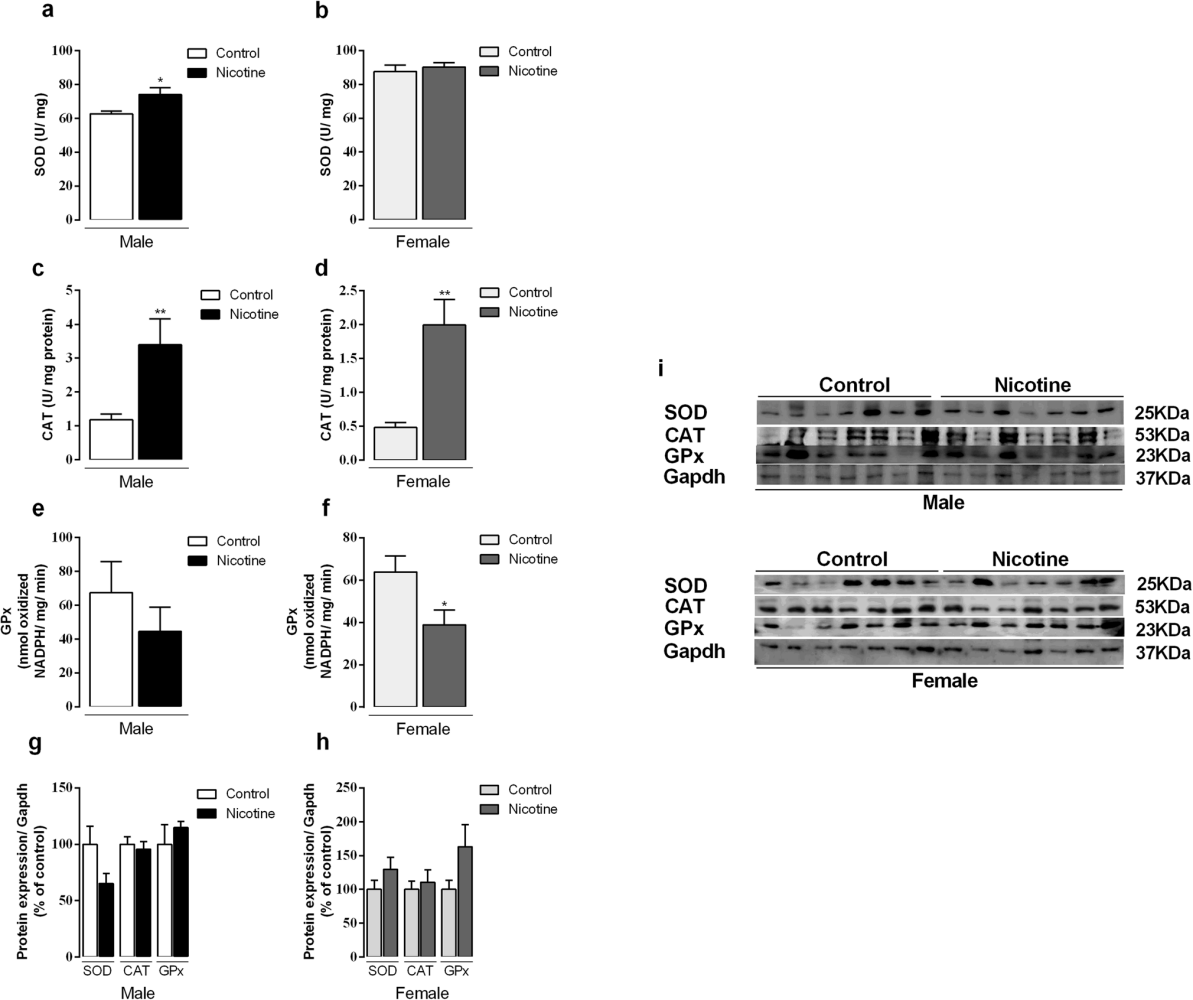
Effects of nicotine exposure during breastfeeding on thyroid activity of antioxidant enzymes: superoxide dismutase (SOD) (**a**, **b**), catalase (CAT) (**c**, **d**) and glutathione peroxidase (GPx) (**e**, **f**) and protein expression (**g**, **h**) in both male and female offspring at 180-day-old. Representative western blots images show all bands and cropped membrane in specific molecular weight (**i**). Data are expressed as mean ± S.E.M, n = 6–7 animals from different litters/group; *p < 0.05, **p < 0.001.

We also evaluated the main enzymes of antioxidant system in the thyroid of adult offspring. SOD activity was higher (+ 18%, *p* = 0.02) only in NIC males (Fig. 4a,b, p < 0.05). CAT activity was higher in both male and female NIC groups (3- and 4-fold, respectively, *p* < 0.05) (Fig. 4c,d), whereas GPx activity diminished only in NIC females (− 40%, *p* = 0.04) (Fig. 4e,f). Protein expression of SOD, GPx and CAT did not change in any groups (Fig. 4g,h). Representative western blot bands for each protein are shown in Fig. 4i.

### Oxidative stress biomarkers

**Figure 5:**
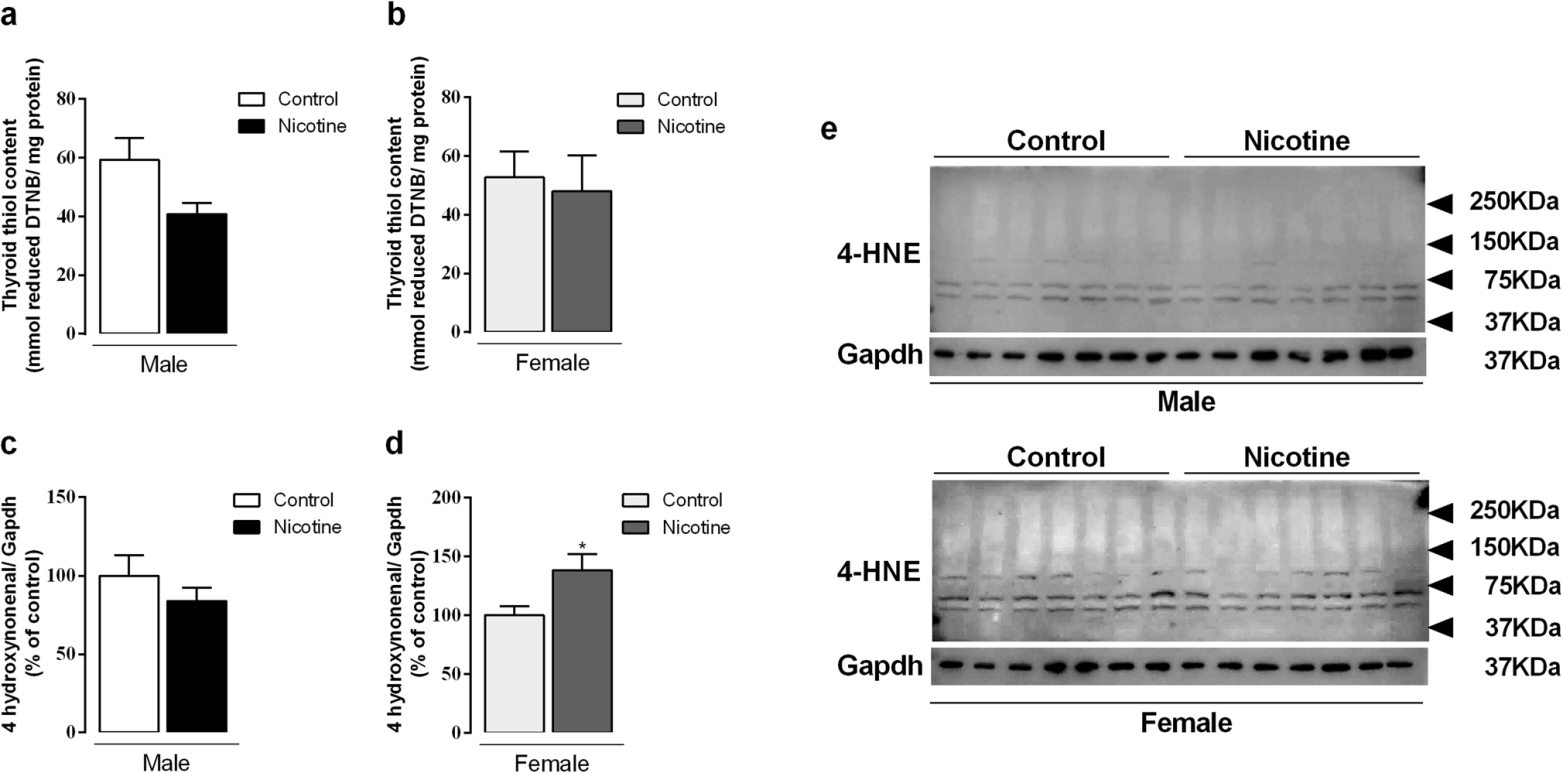
Effects of nicotine exposure during breastfeeding on thyroid biomarkers of oxidative stress: thiol content (**a**, **b**) and 4-HNE (**c**, **d**) in both male and female offspring at 180-day-old. Representative western blots images show all bands and cropped membrane in specific molecular weight (**e**). Data are expressed as mean ± S.E.M, n = 6–7 animals from different litters/group, *p < 0.05.

Thiol content did not change in both males and females (Fig. 5a,b). 4-HNE protein expression only increased in NIC female offspring (+ 38%, *p* = 0.03) (Fig. 5c,d). Representative western blot bands for each protein are shown in Fig. 5e.

### AR and ER protein expression

**Figure 6:**
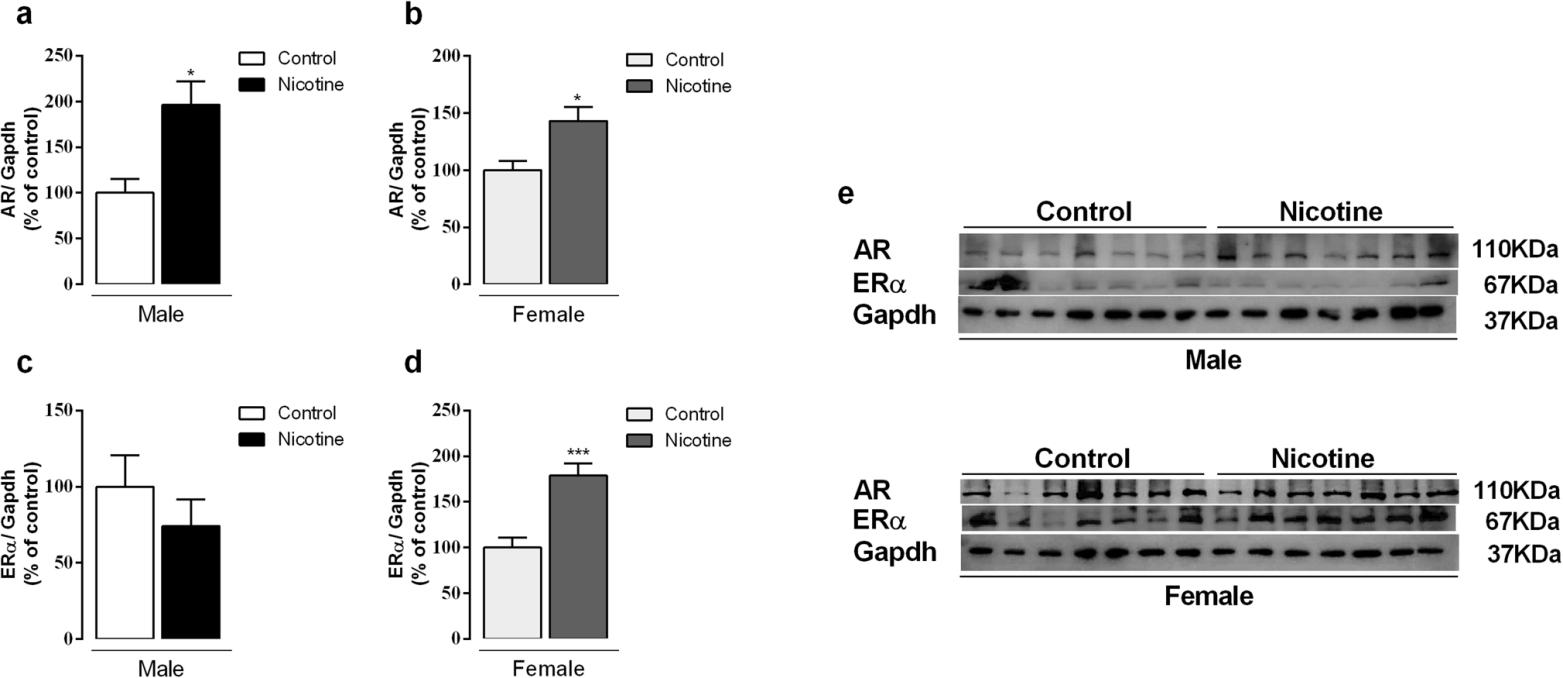
Effects of nicotine exposure during breastfeeding on thyroid AR (**a**, **b**) and ERα (**c**, **d**) protein expression in both male and female offspring at 180-day-old. Representative western blots images show all bands and cropped membrane in specific molecular weight (**e**). Data are expressed as mean ± S.E.M, n = 7 animals from different litters/group; *p < 0.05, ***p < 0.001.

Male and female NIC offspring had higher AR protein expression (+ 96% and + 42%, respectively, *p* < 0.05) when compared to control ones (Fig. 6a,b). NIC males did not show changes in ERα (Fig. 6c), while NIC females had higher ERα protein expression (+ 78%, *p* = 0.0005) (Fig. 6d). Representative western blot bands for each protein are shown in Fig. 6e.

### Thyroid morphology

**Figure 7:**
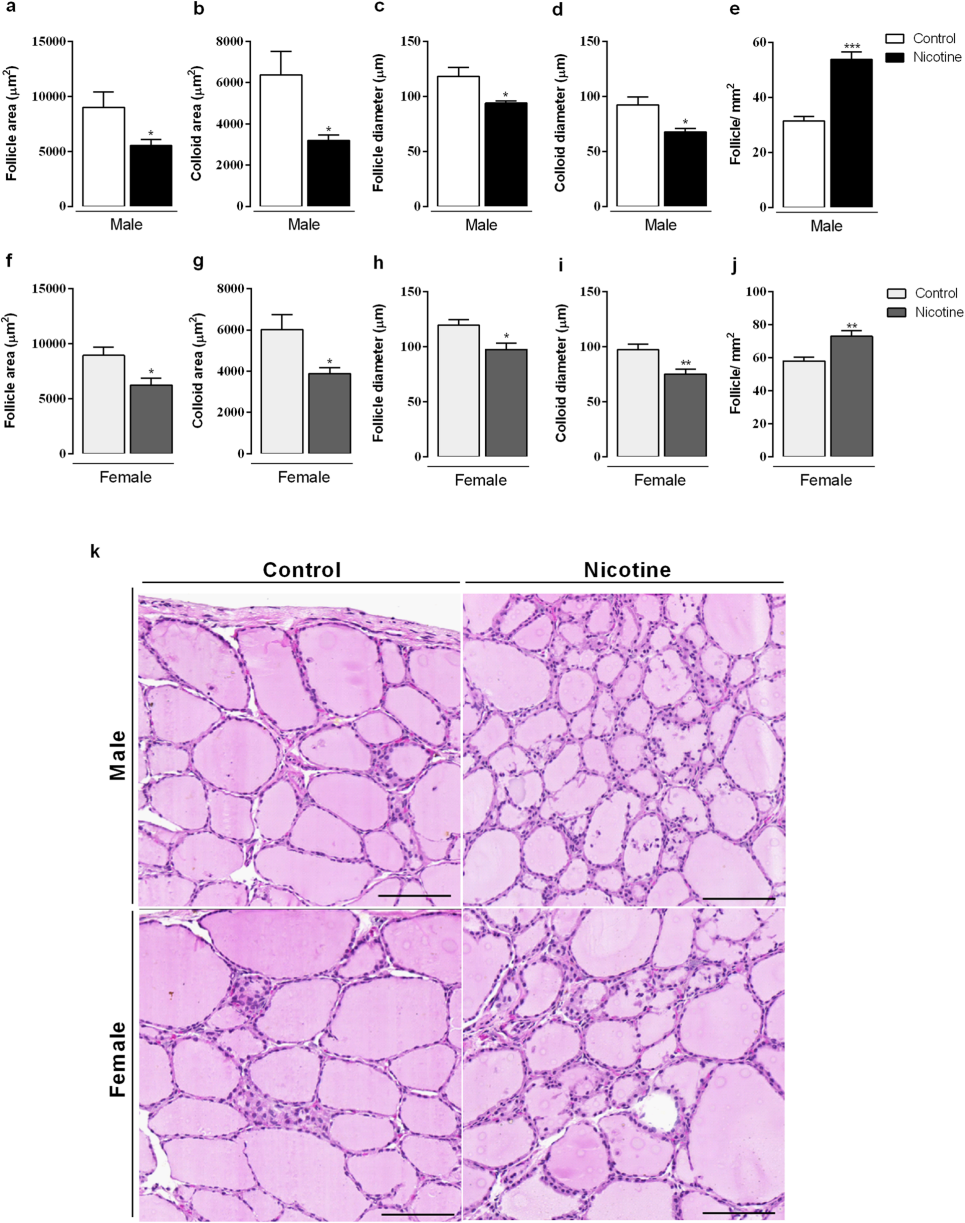
Effects of nicotine exposure during breastfeeding on thyroid morphology: follicle area (**a**, **f**), colloid area (**b**, **g**), follicle diameter (**c**, **h**), colloid diameter (**d**, **i**) number of follicles (**e**, **j**) in both male and female offspring at 180-day-old. Photomicrographs of thyroid gland histological sections stained in hematoxylin and eosin (H&E) are shown (**k**). Magnification × 20, scale bar 50 μm, n = 5 animals from different litters/group, *p < 0.05.

Male and female NIC offspring displayed similar thyroid morphology, with lower follicle area (*p* < 0.05, Fig. 7a,f), colloid area (*p* < 0.05, Fig. 7b,g) as well as lower follicle and colloid diameters (*p* < 0.05, Fig. 7c,d,h,i). However, the number of follicles was higher when compared with the controls (*p* < 0.05, Fig. 7e,j). NIC offspring from both sexes did not show a single epithelial layer; there was an accumulation of epithelial cells between the follicles. Photomicrographs of thyroid gland histological sections stained in hematoxylin and eosin (H&E) are shown in Fig. 7k.

## Discussion

This study demonstrated for the first time that maternal nicotine exposure during breastfeeding leads to thyroid redox imbalance in offspring of both sexes. Male NIC offspring had higher body mass, low TSH, T_3_, T_4_ and testosterone plasma levels; thyroids displayed higher NIS protein expression, low TPO activity and higher SOD and CAT activities, and higher AR protein expression. In contrast, female NIC offspring displayed higher T_3_ and T_4_ plasma levels; thyroids had higher TSHr and TPO protein expression, higher CAT and lower GPx activities, accompanied by higher 4-HNE, AR and ERα protein expressions.

Epidemiological and experimental studies have shown that prenatal nicotine exposure induces obesity and increases the risk of diseases later in life^18^. Nicotine exposure, specifically during lactation, compromises milk yield^19^ and induces adaptive changes in the offspring that lead to metabolic disorders, which include: overweight, insulin and leptin resistance and thyroid dysfunction^9,10,20^. The evaluation of thyroid hormones status in male offspring from nicotine exposed dams has already been reported by our group in 180-day-old rats^9^. These animals were programmed for central hypothyroidism, which was confirmed here. On the other hand, we demonstrated here, for the first time, that NIC females at 180-day-old display higher T_3_ and T_4_ plasma levels, with no effects on TSH. This sexual dimorphism could be explained, at least in part, by thyroid hypofunction in males and hyperfunction in females. Due to this interesting finding, we performed some evaluations as to understand the thyroid function in this programming model, focusing on the hormonogenesis process.

Thyroid hormones production depends on many steps. One of the most important one is the transport of I^−^ across the membrane of the thyroid follicular cells by NIS. In our study, NIC males displayed higher NIS protein expression, despite low TSH, which is a major stimulator of NIS expression^21^. As NIS activity was not measured, this can be considered a limitation of this study. Despite the reported antithyroid effects of smoking, it seems that it is due mainly to thiocyanate effect on thyroid I^−^ uptake^22^. Until now, there were no studies addressing the effect of nicotine on NIS. Conversely, NIC females did not show changes in NIS protein expression as well as in TSH level. Concerning the higher thyroid hormone levels in NIC females, we suggest an increase of TSH action, since TSHr is overexpressed in the thyroid gland. Also, increased T4 and T3 can be due to a decrease in peripheral deiodination. Thus, the evaluation of hepatic D1 in NIC females could help understanding the origin of higher circulating thyroid hormones.

At the apical membrane of follicular cells, I^−^ is rapidly oxidized by TPO. Our data demonstrated that nicotine exposure during breastfeeding decreased TPO activity in the adult male rat offspring, but did not modify its protein expression. These findings are coherent with the decreased levels of thyroid hormones in plasma. Differently, in NIC females, TPO activity did not change notwithstanding this protein overexpression, which could be due to a higher estrogen effect on the thyroid gland, since ERα was increased despite the normal estradiol plasma levels. In fact, estradiol regulates TPO expression and activity as well as NIS in the thyroid gland. Lima et al.^23^ demonstrated, in adult intact and ovariectomized animals, that high doses of estradiol increased TPO activity, suggesting a direct regulation of TPO activity by estrogen in the thyroid gland, independent of changes in TSH level.

High levels of ROS impact the oxidative balance of thyroid glands, which can potentially damage thyrocytes macromolecules, leading to thyroid diseases^15^. ROS is normally maintained at low intracellular concentrations due to the action of peroxidases, such as GPx and CAT, as well as of other antioxidant mechanisms^14^. In our study, H_2_O_2_ generation was not different among groups, but our analysis was restricted to NADPH oxidases. SOD and CAT activities were higher in the thyroid of NIC male offspring. On the other hand, NIC female offspring had higher CAT activity, but lower GPx activity. Interestingly, 4-HNE levels, a marker of oxidative stress^24^, were higher in the thyroids of NIC female progeny. Taken together, these results suggest that the female offspring thyroid is subjected to an oxidative stress that was not observed in males, probably due to the observed changes in antioxidant defense and the hyperthyroidism status. It has already been reported that TSH regulates antioxidant enzymes in the thyroid gland. In hyperthyroidism, for example, it was observed increased oxygen consumption, mitochondrial dysfunction and increased markers of oxidative stress accompanied by decreased antioxidative capacity^25,26^. Diana et al.^27^ confirmed this hypothesis by stimulating TSHr in human thyroid culture cells with autoantibodies. TSHr stimulation increased in vitro SOD release and the 4-HNE, which was confirmed by the in vivo measurements showing higher oxidative stress markers, such as malondialdehyde, 8-isoprostane and 8-hydroxy-2-deoxy guanosine in the urine of patients with untreated Graves’ disease. In contrast, blocking human TSHr did not show any effect. Considering that, despite having normal TSH levels, NIC females showed increased TSHr expression, we suggest that the overstimulation of this receptor could regulate the redox system in the thyroid gland. To our knowledge, the literature neither reports any evidence regarding nicotine effect on thyroid gland redox imbalance, nor does it report its association with metabolic programming. Given the current findings, other oxidative stress markers should also be studied to better understand the oxidative damage status in this experimental model.

Concerning steroids measurements, NIC male progeny showed lower plasma testosterone and increased thyroid AR protein expression, possibly to compensate the decreased hormone levels. High testosterone levels are correlated with hyperthyroidism in men; it is conceivable that testosterone modulates key enzymes involved with thyroid hormonogenesis, such as TPO^28^. This association was found here, since NIC males have low testosterone and show hypothyroidism. On the other hand, despite the fact that NIC females did not show altered estradiol level, they had higher ERα protein expression in the thyroid gland. These findings, which were accompanied by a redox imbalance, suggest that nicotine exposure during breastfeeding results in a permanent imprinting of the thyroid glands, rendering females more susceptible to the development of thyroid diseases. In humans, it has already been reported that the prevalence of thyroid diseases is higher in women than in men^29^. In fact, estrogens affect thyroid function both directly and indirectly, as demonstrated by others^30–32^. Estrogen acts increasing iodide uptake, TPO activity and TG expression, and it modulates TSH level. Furthermore, estrogen also influences the thyroid gland redox status, as previously reported^17,33^. It is conceivable that the mechanisms underlying this influence involve the estrogen-regulated NOX4 and DUOX2 activity and expression. The existence of a sex-dimorphism in the thyrocyte redox balance due to higher NOX4 expression and decreased enzymatic antioxidant defense was observed in the thyroids of adult female rats^34^. Although we did not evaluate the expression of these enzymes, we suggest that they could be altered in NIC female offspring, considering the ERα overexpression observed in these animals. Contrary to our previous findings in other tissues obtained from NIC female offspring, in the specific case of the thyroid gland, females do not seem to be protected by estrogen as evidenced by the metabolic programming, demonstrating a sex- and tissue-dependent phenomenon.

Regarding thyroid morphology, both NIC males and females displayed a similar phenotype, which include lower follicle and colloid area/diameter accompanied by a greater number of follicles with irregular epithelium. Thyroid dysfunction is characterized by changes in thyroid morphology. In the rat hypothyroidism, different sizes of altered follicles with variable quantity of little dense colloid^35^ can be observed. In contrast, in the mice hyperthyroidism, there are a prevalence of follicles with different sizes, containing a large quantity of colloid, differentiated epithelium and smaller thyrocytes^36^. Despite the morphological similarities between sexes, interestingly, the thyroid status differs between males and females, a finding that cannot be directly associated with thyroid follicle changes.

Taken together, the present results indicate that nicotine exposure during the breastfeeding period programs the rat offspring to a redox dyshomeostasis in the thyroid gland that directly impairs thyroid morphology and hormone synthesis at adulthood in a sex specific manner. In addition, AR and ER in the thyroid gland could potentially contribute to the sexually dimorphic dysfunctions observed in this model.

## Methods

### Ethics, animals and experimental groups

The Ethical Committee for Use of Laboratory Animals of the Biology Institute, Rio de Janeiro State University (CEUA/033/2017) previously approved all experimental procedures. All experiments were performed in accordance to the American Physiological Society’s guiding principles^37^. Throughout the experiment, all animals were housed under controlled conditions in a 12-h light–dark cycle (lights on from 7 a.m. to 7 p.m.) and at a temperature of 21 ± 2 °C. Water and a standard rodent chow diet (Nuvilab, São Paulo, Brazil) were offered ad libitum.

Three months old female and male *Wistar* rats were mated and, upon detection of pregnancy, the pregnant rats were housed in individual cages. After birth, all litters were normalized to six pups per litter. Two days after birth, lactating rats (n = 15 dams/group) were randomly assigned to one of the following groups: (a) nicotine (NIC)—dams were anesthetized with thiopental (ip 30 mg/kg of body mass). A 3 × 6 cm area on the back was shaved and an incision was made to allow for the s.c. insertion of osmotic minipumps (Alzet, 2ML2, Los Angeles, CA, USA). Pumps were prepared with nicotine free-base (Sigma, St Louis, MO, USA) diluted in NaCl 0.9% solution to release a dose of 6 mg/kg of nicotine/day for 14 days (from the 2nd to 16th day of the lactation period), as previously described^9^; (b) control—dams were implanted with osmotic minipumps containing only saline solution. We chose to perform nicotine exposure via subcutaneous osmotic minipumps to avoid the adverse effects of nicotine peaks. In our rat model, the regimen of maternal nicotine exposure (total of 84 mg/kg in 14 days per dam) approximates that of moderate to heavy human smokers^38^. Offspring were exposed to nicotine exclusively via milk and at weaning; the blood cotinine in the pup was 20 ng/ml^20^.

At 180-day-old, offspring were weighed and anesthetized with thiopental (ip 150 mg/kg of body mass) and euthanized by cardiac puncture to obtain blood. Blood samples were collected in a heparin tube, centrifuged (1260 × *g*, 25 min, 4 °C) and then stored at − 20 °C for posterior analyses. Thyroid glands were collected, weighed and stored at − 80 °C for analyses.

### Plasma analysis

TSH was measured by specific rat Elisa Kit (Alpco Diagnostics, NH, USA). The intra-assay variation was 5.9%, with 0.1 ng/ml as the lower limit of detection. Total T_3_ and free T_4_ were determined by radioimmunoassay (RIA), using a commercial kit (MP Biomedicals, LLC, NY, USA), with the range of detection between 50 and 800 ng/dl and 0.3 and 11 ng/dl, respectively. Intra-assay variations were 2.9% (T_4_) and 3.5% (T_3_). Testosterone and estradiol were evaluated by RIA kits (MP Biomedicals, LLC, NY, EUA). The sensitivities of the assays were 0.1 ng/ml and 10 pg/ml, respectively. Intra-assay variations were 1.5% (testosterone) and 1.6% (estradiol).

### Western Blotting

Thyroid glands were collected and frozen in liquid nitrogen and subjected to maceration in an extract buffer (T-PER Tissue Protein Extraction) containing a protease inhibitor cocktail (Roche). Western blotting technique followed the protocol previously described in Miranda et al.^39^ with some adaptations. The homogenates were centrifuged at 15,294 × *g* for 20 min at 4 °C (Eppendorf 5417R, Hampton, USA). Total protein content was determined using a BCA Protein Assay Kit (Thermo Scientific, Rockford, IL, USA). Samples were treated with Laemmli sample buffer^40^ (w/v: glycerol, 20%; β-mercaptoethanol, 10%; 10% sodium dodecyl sulfate (SDS), 40%; and 0.5 mol/l Tris at pH 6.8, 0.5%; plus deionized water and bromophenol blue). Total protein extracts (15 µg) were separated by 10% SDS-PAGE at 200 V for 50 min. The proteins were then transferred from the gel to a polyvinylidene difluoride (PVDF) membrane by Trans-Blot turbo system (Bio-Rad Laboratories, Hercules, CA, USA) and blocked with 5% BSA in Tween-Tris-buffered saline (TTBS; Tris–HCl, 1 mol/l; NaCl, 5 mol/l; and Tween 20, 0.05%, v/v) for 90 min with continuous shaking. Membranes were incubated overnight with primary antibodies described in Table 2. PVDF membranes were washed three times (5 min) with Tween–TBS (0.1%), followed by 1 h incubation with appropriate biotin-conjugated secondary antibody (Table 2). Then, membranes were washed and incubated 1 h with streptavidin–horseradish peroxidase conjugate (RPN1231V; GE Healthcare, Buckingham, Shire, UK). Immunoreactive proteins were visualized with chemiluminescent western blotting substrate (Clarity, Bio-Rad Laboratories, Hercules, CA, USA) using an Image Quant LAS (GE Healthcare, Buckingham, Shire, UK) in a single automatic exposure. Bands were quantified by densitometry using Image J 1.4 software (Wayne Rasband, National Institutes of Health, Bethesda, MA, USA). Cyclophilin or glyceraldehyde 3-phosphate dehydrogenase (Gapdh) protein content was used as loading control. The membranes were cropped following the molecular weight pattern of each protein of interest. Each cropped membrane was incubated with a specific antibody for detection of each protein that was in different molecular weights. Representative western blots images show all bands (n = 7/group) and cropped membrane in specific molecular weight (see Supplementary information).

**Table 2:** Antibodies used for western blotting.

Primary antibodies			Secondary antibodies		
Antibody	Catalogue number/distributed by	Dilution	Catalogue number/distributed by	Dilution	Specificity
NIS	NBP1-70342/Novus Biological Centennial, CO, USA	1:1000	B8520/Sigma-Aldrich MO, USA	1:10,000	Anti-mouse
TPO	sc-58432/Santa Cruz Biotechnology MA, USA	1:1000	B8520/Sigma-Aldrich MO, USA	1:10,000	Anti-mouse
MCT8	sc-47124/Santa Cruz Biotechnology MA, USA	1:500	AP106B/Millipore Corporation CA, USA	1:10,000	Anti-goat
TSHr	sc-53542/Santa Cruz Biotechnology MA, USA	1:500	B8520/Sigma-Aldrich MO, USA	1:10,000	Anti-mouse
Cyclophilin	#51418/Cell Signaling Technology MA, USA	1:1000	B7389/Sigma-Aldrich MO, USA	1:10,000	Anti-rabbit
SOD	sc-133134/Santa Cruz Biotechnology MA, USA	1:500	B8520/Sigma-Aldrich MO, USA	1:10,000	Anti-mouse
CAT	sc-50508/Santa Cruz Biotechnology MA, USA	1:1000	B7389/Sigma-Aldrich MO, USA	1:10,000	Anti-rabbit
GPx	sc-133152/Santa Cruz Biotechnology MA, USA	1:1000	B8520/Sigma-Aldrich MO, USA	1:10,000	Anti-mouse
4-HNE	ab46545/Abcam, MA, USA	1:1000	B7389/Sigma-Aldrich MO, USA	1:10,000	Anti-rabbit
AR	06-680/Millipore Corporation, Temecula, CA, USA	1:1000	B7389/Sigma-Aldrich MO, USA	1:10,000	Anti-rabbit
ERα	06-935/Millipore Corporation, Temecula, CA, USA	1:500	B8520/Sigma-Aldrich MO, USA	1:10,000	Anti-mouse
GAPDH	14C10 #2118/Cell Signaling Technology MA, USA	1:1000	B7389/Sigma-Aldrich MO, USA	1:10,000	Anti-rabbit

*NIS* sodium/iodide symporter, *TPO* thyroid peroxidase, *MCT8* monocarboxylate transporter-8, *TSHr* thyroid-stimulating hormone receptor, *SOD* superoxide dismutase, *CAT* catalase, *GPx* glutathione peroxidase, *4-HNE* 4-hydroxynonenal, *AR* androgenic receptor, *ERα* estrogen receptor alpha, *GAPDH* glyceraldehyde 3 phosphate dehydrogenase.

### Thyroid peroxidase (TPO) activity

TPO activity was evaluated as previously described^41,42^. Thyroids were homogenized in Tris–HCl 50 mM buffer, pH 7.2, containing 1 mM KI. The homogenate was centrifuged at 100,000 × *g*, 4 °C for 35 min. The pellet was suspended in Tris–HCl 50 mM plus triton (0.1% v/v) and incubated at 4 °C for 24 h to solubilize the TPO. The suspension was centrifuged at 100,000 × *g*, 4 °C for 35 min, and the supernatant containing solubilized TPO was used for the activity measurement.

Activity was measured using a mixture containing: 50 mmol/l sodium phosphate buffer, pH 7.4, 24 mmol/l KI, 11 mmol/l glucose, and increasing amounts of solubilized TPO. The reaction was started by the addition of 10 μl of 1 mg/ml glucose oxidase. The increase in absorbance at 353 nm (tri-iodide production) was registered for 5 min on a Hitachi spectrophotometer (U-3300). The ΔA353 nm/min was determined from the linear portion of the reaction curve and related to protein concentration that was obtained by Bradford assay^43^. TPO activity results were expressed as U/μg of protein.

### Thyroid NOX activity

Thyroids were homogenized in a 50 mM sodium phosphate buffer, pH 7.2, containing 0.25 M sucrose, 0.5 mM dithiothreitol, 1 mM ethylene glycol tetra-acid (EGTA), 5 mg/ml aprotinin, and 34.8 mg/ml phenyl methane sulfonyl fluoride (PMSF). First, the homogenates were centrifuged at 600 × *g* for 15 min at 4 °C. To obtain the microsomal fraction, the supernatant was centrifuged twice at 100,000 × *g* for 35 min at 4 °C and the pellets were resuspended in assay buffer (0.5 ml 50 mM sodium phosphate buffer, pH 7.2, containing 0.25 M sucrose, 2 mM MgCl_2_, 5 µg/ml aprotinin and 34.8 mg/ml PMSF). In order to measure H_2_O_2_ generation, the microsomal fraction was incubated in 150 mM sodium phosphate buffer (pH 7.4) containing superoxide dismutase (SOD) (100 U/ml; Sigma, USA), horseradish peroxidase (0.5 U/ml, Roche, Indianapolis, IN), Amplex red (50 µM; Molecular Probes, Eugene, OR), 1 mM EGTA, 1 mM NADPH, in the presence or absence of 1 mM CaCl_2_. Calcium-dependent H_2_O_2_ generation was obtained by subtracting H_2_O_2_ generation in the absence of calcium from that obtained in the presence of calcium. The fluorescence was immediately measured in a microplate reader (Victor X4; PerkinElmer, Norwalk, CT) at 30 °C, using excitation at 530 nm and emission at 595 nm^13^. Specific enzymatic activity was expressed as nmol of H_2_O_2_/h/mg of protein.

### Antioxidant enzyme activities

Thyroid glands of each animal were homogenized in 5 mM Tris HCl, 0.9% NaCl (pH 7.4) containing 0.1 mg/ml aprotinin and 14.3 mM phenylmethanesulfonyl fluoride (PMSF). The homogenate was centrifuged at 720 × *g* for 10 min, 4 °C and the supernatant was used for enzyme activity assays. Total protein content was quantified using a Bradford method^43^. All the enzymatic assays were performed in an UV spectrophotometer (PerkinElmer, LAMBDA, Shelton, CT, USA) at 37 °C. Catalase (CAT) activity was measured according the method previously described^44^. Glutathione peroxidase (GPx) activity was measured by NADPH oxidation at 340 nm^45^ and SOD activity was assayed by the reduction of cytochrome C at 550 nm^46^.

### Thiol content

The thiol residues were determined by reaction with 5,5-dithionitrobenzoic acid (DTNB), cleaving the disulfide bond to give 2-nitro-5-thiobenzoate (NTB^−^), which ionizes to the NTB^2−^ dianion in water at neutral and alkaline pH. NTB^2−^ was quantified in a spectrophotometer by measuring the absorbance at 412 nm and data was expressed as nmol of reduced DTNB/mg protein^34^.

### Morphology

Thyroid samples were fixed in paraformaldehyde 4% for 48 h, followed by dehydration, clarification and inclusion in histological paraplast. The fixed samples were sectioned using a microtome (microTEC Cut4050, Walldorf, Germany) at a thickness of 5 μm. Sections were stained in hematoxylin and eosin (H&E) for morphometric analysis, following standard protocol^47^. Images were obtained using Pannoramic Digital Slide Scanners (Pannoramic MIDI II—3DHISTECH Ltda, Budapest, Hungary.) and then analyzed in a program (CaseViewer 2.3). The diameter of the colloid, the follicle and both areas considered together were analyzed.

### Statistical analyses

Results were expressed as mean ± standard error of the mean (SEM) and analyzed through the statistical program GraphPad Prism 6.0 (San Diego, CA, USA). Data sets were tested for normality using the Kolmogorov–Smirnov test, and the differences between Control and NIC offspring per each sex were analyzed by Student’s *t* test. Statistical difference was considered when *p* < 0.05.

## Supplementary information

Supplementary Figure.

## Data Availability

The datasets generated during and/or analyzed during the current study are available from the corresponding author.
