# Supplementary material for: Thyroid redox imbalance in adult Wistar rats that were exposed to nicotine during breastfeeding
Source: Sci Rep. 2020 Sep 24;10:15646. doi: 10.1038/s41598-020-72725-w (PMC7519108; doi:10.1038/s41598-020-72725-w)

Thyroid redox imbalance in adult Wistar rats that were exposed to nicotine during breastfeeding

Rosiane Aparecida Miranda[1], Egberto Gaspar de Moura[1], Patrícia Novaes Soares[1], Thamara Cherem Peixoto[1], Bruna Pereira Lopes[1], Cherley Borba Vieira de Andrade[2], Elaine de Oliveira[1], Alex C. Manhães[3], Caroline Coelho de Faria[2], Rodrigo Soares Fortunato[2], Patricia Cristina Lisboa[1*]

[1]Laboratory of Endocrine Physiology, Biology Institute, Rio de Janeiro State University, RJ, Brazil; [2]Carlos Chagas Filho Biophysics Institute, Federal University of Rio de Janeiro, Rio de Janeiro, Brazil; [3]Laboratory of Neurophysiology, Biology Institute, Rio de Janeiro State University, RJ, Brazil.

Western blotting images

Males

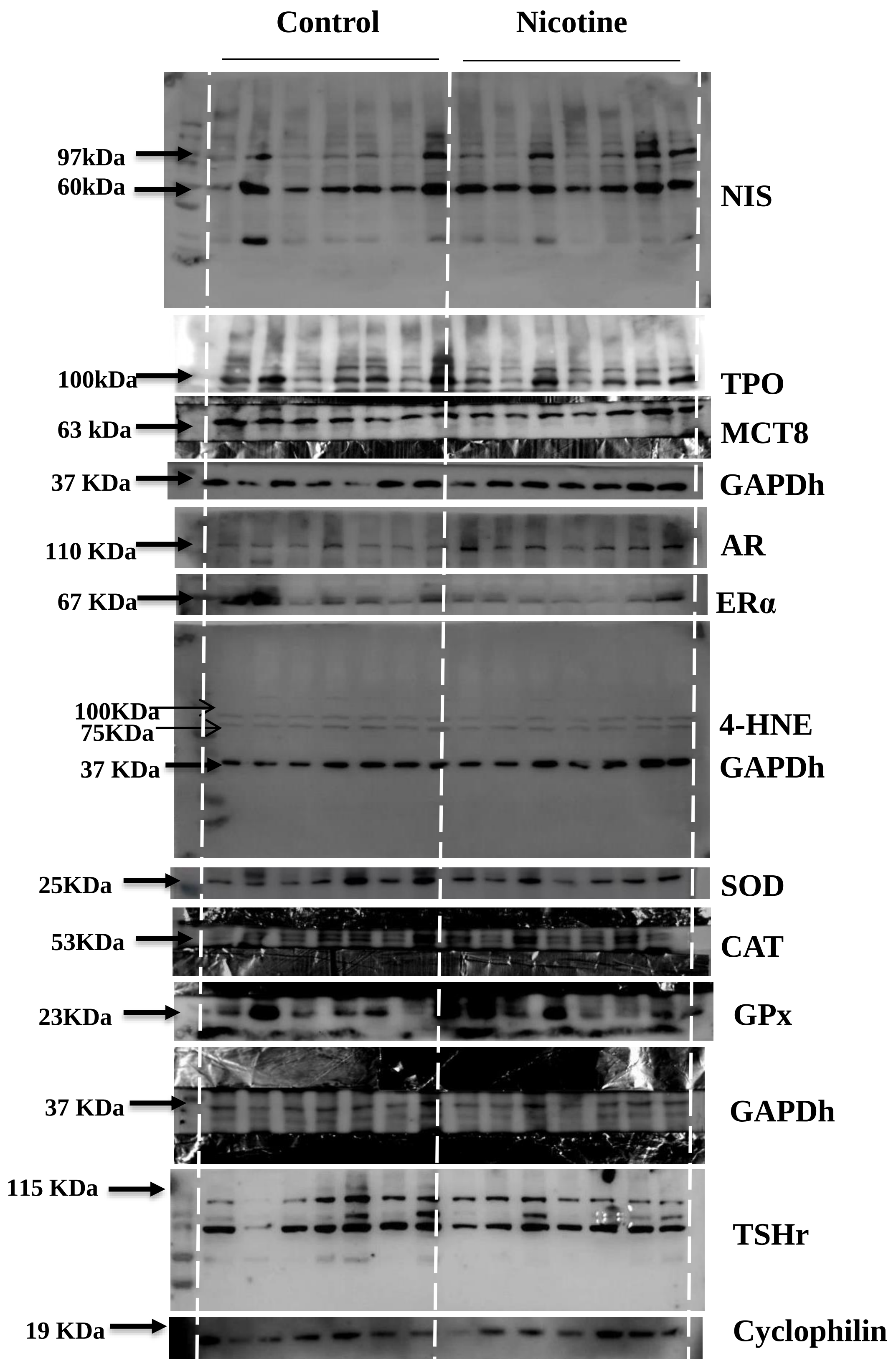

Females

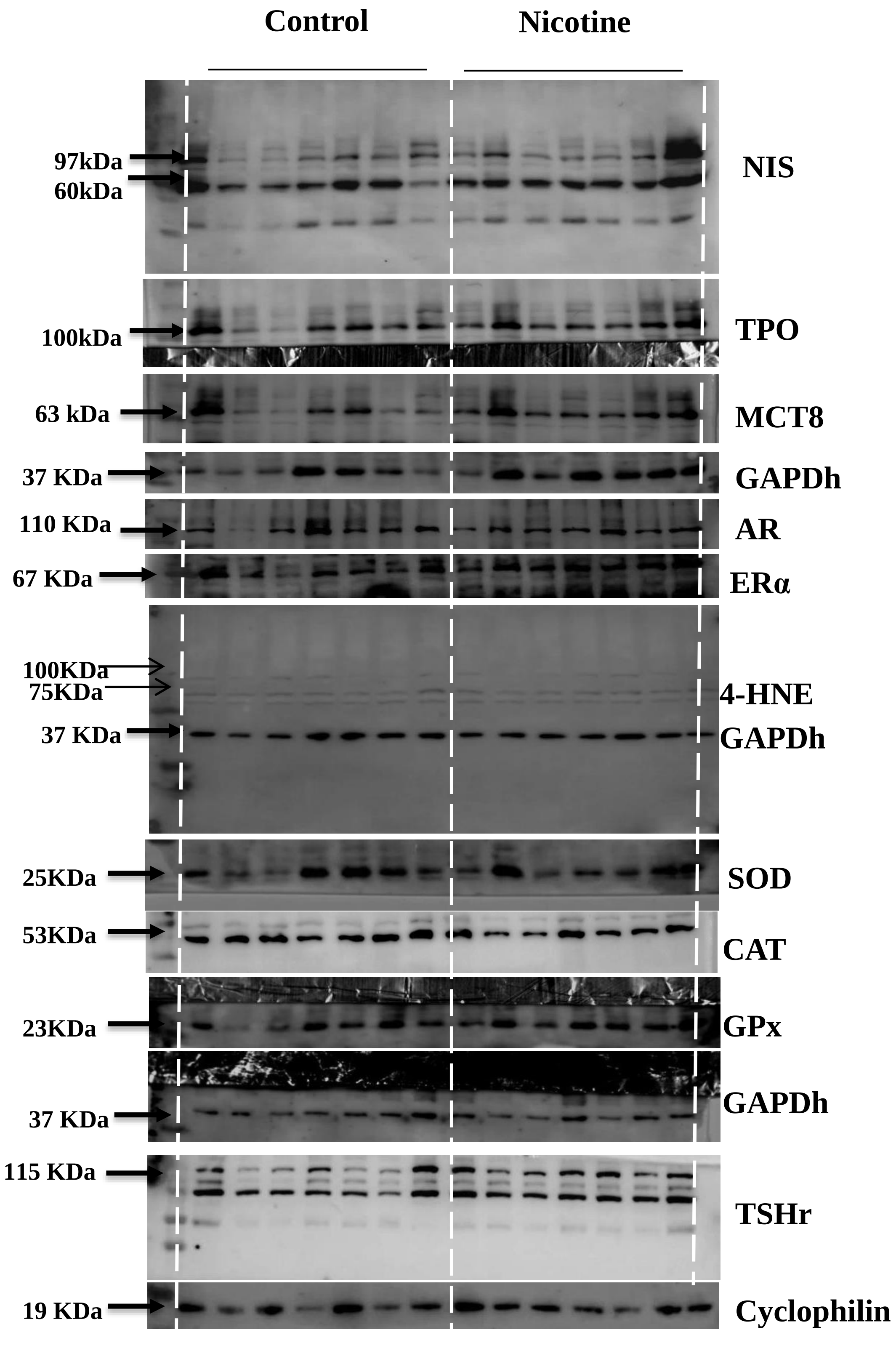

Supplement: Supplementary file 1 — Supplementary Figure. [file 41598_2020_72725_MOESM1_ESM.pdf]
